# (5R)-5-Hydroxytriptolide (LLDT-8) inhibits osteoclastogenesis via RANKL/RANK/OPG signaling pathway

**DOI:** 10.1186/s12906-015-0566-y

**Published:** 2015-03-24

**Authors:** Yi Shen, Ting Jiang, Rongsheng Wang, Shijun He, Mengru Guo, Jianping Zuo, Dongyi He

**Affiliations:** Department of Rheumatology, Shanghai Guanghua Hospital of Integrated Traditional and Western Medicine, Shanghai, 200052 China; Arthritis Institute of integrated Traditional and Western medicine, Shanghai Chinese Medicine Research Institute, Shanghai, 200052 China; Laboratory of Immunopharmacology, State Key Laboratory of Drug Research, Shanghai Institute of Materia Medica, Chinese Academy of Sciences, Shanghai, 201203 China

**Keywords:** (5R)-5-Hydroxytriptolide, Osteoclastogenesis, Osteoprotegerin, RANKL, Rheumatoid arthritis

## Abstract

**Background:**

The aim of this study was to investigate the regulative activity of (5R)-5-hydroxytriptolide (LLDT-8) on receptor activator of nuclear factor κ-B ligand (RANKL)/receptor activator of nuclear factor κ-B (RANK)/Osteoprotegerin (OPG) system in rheumatoid arthritis (RA) and its anti-osteoclastogenesis mechanism.

**Methods:**

The expression of OPG, RANK and RANKL in CD3^+^ T leukomonocytes in both peripheral blood and synovial fluid of RA patients was evaluated by flow cytometry. The levels of interleukin (IL) 1β, IL-6, IL-10, IL-21 and IL-23 in the supernatants of peripheral blood mononuclear cells (PBMCs) and synovial fluid mononuclear cells (SFMCs) were assayed by ELISA. Tartaric acid phosphatase (TRAP) staining was used to identify the osteoclast-like cells derived from RAW264.7. Western blotting analysis was used to check the downstream molecules of RANKL.

**Results:**

LLDT-8 increased the rate of OPG expression in CD3^+^ T leukomonocytes in peripheral blood as well as the ratio of OPG/RANKL in both peripheral blood and synovial fluid. LLDT-8 inhibited IL-1β, IL-6, IL-21 and IL-23 secretion, but promoted the secretion of IL-10 in the supernatants of PBMCs and SFMCs. In addition, LLDT-8 decreased the number of TRAP-positive cells derived from RAW264.7 in the presence of RANKL and M-CSF. Furthermore, LLDT-8 also inhibited the expression of p-IκB, a key regulator of RANKL signaling pathway.

**Conclusions:**

LLDT-8 exerts its anti-osteoclastogenesis effect in RA probably through regulating RANKL/RANK/OPG system and its downstream signaling pathway as well as cytokine productions.

## Background

Rheumatoid arthritis (RA) is a kind of autoimmune disease, which usually results in a chronic, systemic inflammatory disorder that may affect many tissues and organs, especially joints. RA is characterized by inflammatory synovitis and destruction of cartilage and bone, which usually leads to substantial loss of function and mobility. How to delay with or prevent bone destruction caused by RA, and improve the life quality of patients has been the ultimate goal of RA treatment and nursing [1]. Tripterygium Wil’fordii Hook.F, belonging to plants of the genus Euonymus Corey Gong, is a traditional Chinese medicine, the extracts of which have a variety of pharmacological effects, such as anti-inflammatory, antibiosis, antifertility, immunosuppression, anti tumor and etc. Thus, they are widely used in the treatment of rheumatic disease, autoimmune disease, organ transplantation, nephroma, asthma and tumor [2]. Among the extracts, Ttriptolide is the most active compound, which has strong immunosuppressive activity. However, Ttriptolide could not be directly used in clinic because of its biological toxicity and narrow therapeutic window [3]. (5R)-5-hydroxytriptolide (LLDT-8) is a new analog of Ttriptolide, which has been qualified and optimized in structure, and shows lower cytotoxicity and relatively higher immunosuppressive activity. In our previous study, we found that the toxicity of LLDT-8 is significantly reduced compared with triptolide. General speaking, it showed a 122-fold lower cytotoxicity in vitro and 10-fold lower acute toxicity *in vivo* [4]. Previous studies also demonstrated that LLDT-8 has a variety of immunosuppressive activities and significant therapeutic effects *in vitro* and *in vivo* [5-7]. However, how LLDT-8 influences osteoclastogenesis is still unknown.

The purpose of our study was to assess the effects of LLDT-8 on RANK/RANKL/OPG signaling pathway and osteoclastogenesis. Our work suggested that LLDT-8 up-regulated OPG expression in CD3^+^T leukomonocytes in peripheral blood as well as the ratio of OPG/RANKL in both peripheral blood and synovial fluid, and it also inhibited inflammatory cytokine secretion in the supernatants of PBMCs and SFMCs. Besides, LLDT-8 may reduce osteoclastogenesis by inhibiting NF-κB signaling. These results guarantee a good prospect in further clinical tests of LLDT-8 for its therapeutic potential in the treatment of RA.

## Methods

### Human sample

Thirty two specimens in this study were obtained from Shanghai Guanghua Hospital, among which 21 specimens were peripheral blood and 11 specimens were synovial fluid. The study was approved by the Research Ethics Board of Shanghai Guanghua Hospital, and written informed consents were obtained from all patients.

The 32 specimens were from 29 RA patients, and 27 were female. Patients aged 55.55 ± 12.43 years old, and the duration of illness were in the span of 11.23 ± 9.82 years. All the patients fulfilled the ACR 1987 revised classification criteria for RA. Disease activity score in 28 joints (DAS28), which recommended by European League Against Rheumatism (EULAR), was used to evaluate the disease activity of RA, and 28 patients were in disease activity (DAS28 ≥ 2.6) (Table 1). Besides, 28 patients had taken drugs which used to treat RA before the specimens were obtained (Table 2).

**Table 1:** **Disease activity evaluated by DAS28**

**Disease activity**	**The number of cases**
Remission	1
Low	0
Moderate	9
High	19

DAS28(ESR) = 0.56*(TJC28) + 0.28*(SJC28) + 0.014*VAS + 0.70*ln(ESR). TJC, means the number of tender joints of 28 counted; SJC, means the number of swollen joints of 28 counted; VAS, the score which scaled out by the subject on 100 mm Visual analog scale to match with joint pain [8]. DAS28 < 2.6, indicated Remission; 2.6 ≤ DAS28 < 3.2, indicated low disease activity; 3.2 ≤ DAS28 ≤ 5.1, indicated moderate disease activity; DAS28 > 5.1, indicated high disease activity [9].

**Table 2:** **Medications of RA patients before the specimens were obtained**

**Drugs**	**The number of cases**
No treatment	1
Taking non-steroidal anti-inflammatory drugs	28
Taking 1 disease modifying antirheumatic drug	4
Taking 2 disease modifying antirheumatic drug	17
Taking ≥ 3 disease modifying antirheumatic drug	4
Taking glucocorticoid	13
Taking biologics	3

Disease modifying antirheumatic drugs include methotrexate, leflunomide, sulfasalazine, penicillamine, hydroxychloroquine, and some herb extracts such as total glucosides of paeony, tripterygium glycosides and the extract of Caulis Sinomenii. Dose of glucocorticoid equals to prednisone 2–10 mg daily. Biologics include Etanercept (2 cases) and Infliximab (1 case).

### Cell line

Murine RAW264.7 cell line was obtained from Chinese Academy of Sciences, Shanghai Institute of Materia Medica (Shanghai, China).

### Reagents

LLDT-8 was obtained from Shanghai Pharmaceutical Group (Shanghai, China). RPMI 1640 medium and DMEM were purchased from Hyclone (USA). Fetal bovine serum was purchased from Gibco (USA). Lymphocyte separation medium was purchased from MP Biomedicals (USA). TRAP staining kit was purchased from Sigma-Aldrich (USA). Rat soluble RANK ligand and rat M-CSF were purchased from Peprotech (USA). Antibodies specific for phospho-IκB, phospho-p38, phospho-JNK, phospho-ERK and phospho-Akt were purchased from Cell Signaling Technology (USA).

### PBMC and SFMC isolation

Aseptic and heparin anticoagulant peripheral blood or synovial fluid was prepared, and two-fold diluted by PBS, then transferred to 15 ml centrifuge tube with LSM, and centrifuged at 2500 rpm for 20 min at room temperature. The white mononuclear cells were aspirated, and then washed with PBS (at 2000 rpm for 10 min). The cell pellets were collected, and diluted to suitable concentration with culture medium (RPMI 1640 with 10% FBS).

### Flow cytometry

PBMCs or SFMCs were separated, and seeded at a density of 1 × 10^6^ cells/well in 96-well plates in the presence of LLDT-8 (0, 25 and 50 nM, respectively) for 24 hours in incubator. The cells were harvested and washed with FACS washing buffer followed by incubation with anti-human CD3 (eBioscience, USA) and OPG (bony-1, SANTA CRUZ Biotec, USA) at 4°C for 20 min. After washed by FACS washing buffer, the cells were incubated with goat anti-rat IgG-FITC (SANTA CRUZ) at 4°C for 20 min, then PE conjugated anti-human RANK (R&D Systems, USA), APC conjugated anti-human RANKL (R&D Systems) and Percp conjugated anti-human CD3 (Miltenyi Biotec, Germany) were added respectively, and incubated at 4°C for 20 min. Then the cells were detected by flow cytometry.

### ELISA

PBMCs or SFMCs were separated, and seeded at a density of 1 × 10^6^ cells/well in 96-well plates in the presence of anti-human CD3 (0.4 μg/ml) and LLDT-8 (0, 12.5, 25, and 50 nM, respectively) for 48 hours in incubator. The cell culture supernatants were harvested and the levels of cytokines (IL-1β, IL-6, IL-21, IL-23 and IL-10) were assayed by ELISA kits (eBioscience). All the processes were followed according to the manufacturer’s instructions. In the end a standard curve from the data produced from the serial dilutions with concentration on the X axis (log scale) *vs* absorbance on the Y axis (linear) was drawn to calculate the concentration of the protein.

### Osteoclast formation

RAW264.7 cells were grown in DMEM supplemented with 10% FBS and 1% penicillin/streptomycin. For differentiation of osteoclasts, RAW264.7 cells were seeded at a density of 2 × 10^4^ cells/well in 24-well plates, and cultured in the presence of RANKL (50 ng/ml), M-CSF (50 ng/ml) and LLDT-8 (0, 12.5, 25 and 50 nM, respectively) for 6 days. The culture medium was replaced every 3 days. The cells were stained by TRAP kit according to the manufacturer’s instructions on day 1, 2, 3 and 6. Osteoclast formation was determined to be TRAP-positive staining multinuclear cells using light microscopy. The total number of TRAP-positive cells in each well was counted.

### Western blotting analysis

RAW264.7 cells were seeded at a density of 1 × 10^7^ cells/well in 6-well plates in DMEM supplemented with 10% FBS and 1% penicillin/streptomycin. After cell attachment, the culture medium was replaced by DMEM supplemented with LLDT-8 (0 and 50 nM, respectively) for 4 hours, then the cells were induced by culture medium supplemented with 50 ng/ml RANKL for 0, 10, 30 and 60 min, respectively. The cells were collected and lysed by 1 nM PMSF SDS on ice for 30 min. The cellular lysates were loaded, and proteins were separated on SDS-PAGE and transferred to nitrocellulose filter. The blots were blocked with 5% BSA TBST for 1 h at room temperature, then probed with rabbit anti-mouse antibodies against p-IκB, p-P38, p-JNK, p-ERK, p-Akt (1:1000) and GAPDH (1:6000) overnight at 4°C. After five washes, the blots were subsequently incubated with a HRP-linked secondary antibody (1:2500) for 90 min at room temperature. The blots were visualized by ECLTM Prime Western Blotting Detection Reagent. The pictures were adorned by ImageQuant software (Photoshop CS2).

### Statistics

The values given are mean ± SEM. The difference between the experimental groups and the control was assessed by one-way analysis of variance (ANOVA) with LSD test. The difference is significant if *p* < 0.05.

## Results

### LLDT-8 up-regulated OPG expression on CD3^+^ T leukomonocyte, and increased the ratio of OPG to RANKL

**Figure 1:**
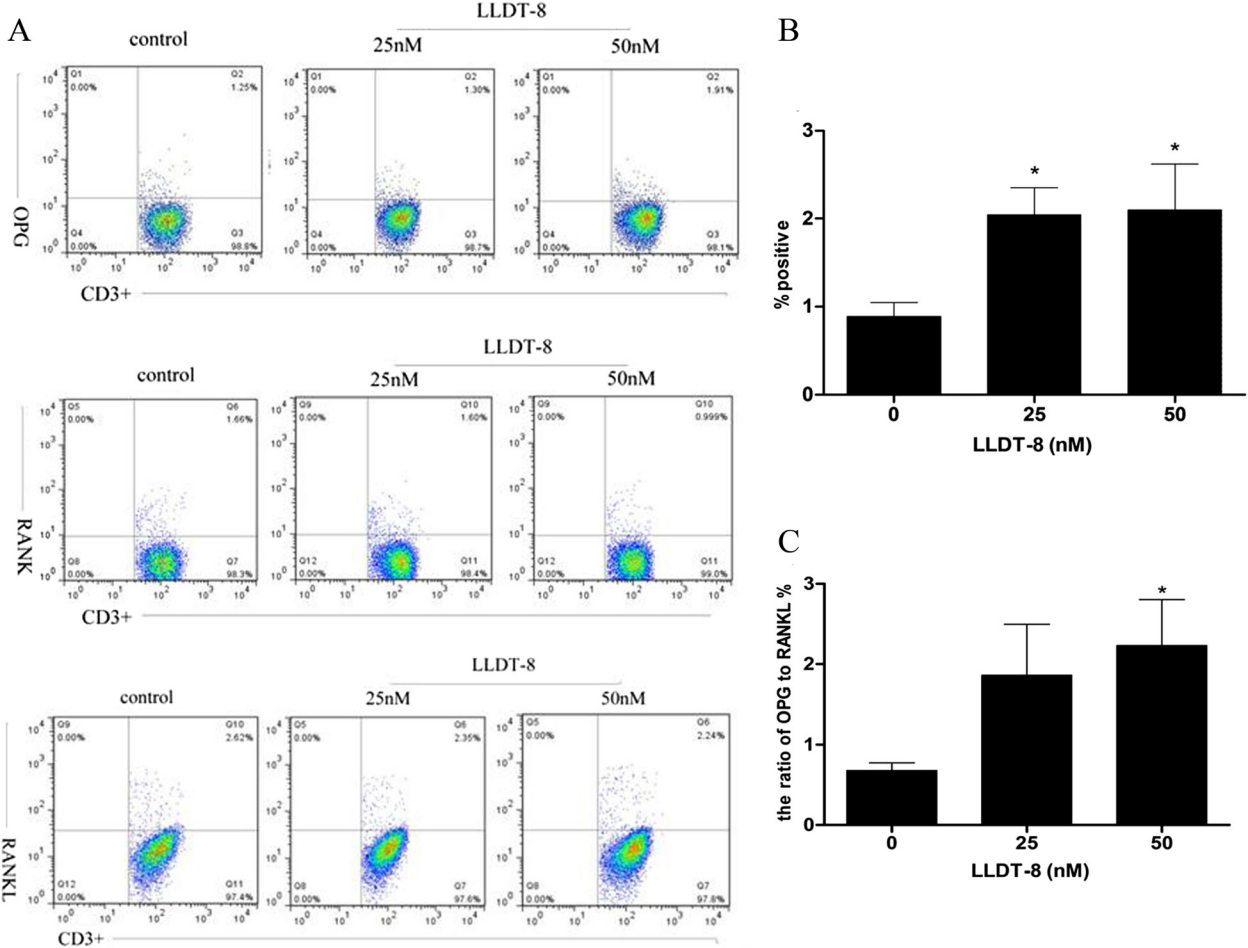
**LLDT-8 up-regulated OPG expression and increased the ratio of OPG/RANKL on CD3** ^**+**^ **T leukomonocyte in peripheral blood of RA patients.** PBMCs were isolated from peripheral blood of RA patients and treated with various concentrations of LLDT-8 (0, 25 nM and 50 nM, respectively) for 24 hours, then stained and detected by flow cytometry. **(A)** The psuedocolor plots of OPG, RANK and RANKL. **(B)** The rate of OPG expression on CD3^+^ T leukomonocyte in the three groups. **(C)** The ratio of OPG to RANKL on CD3^+^ T leukomonocyte in the three groups. *p < 0.05 compared to the control group (0 nM).

We isolated PBMCs from blood sample of RA patients, incubated them with various concentrations of LLDT-8 (0, 25 nM and 50 nM) for 24 hours, and examined the OPG, RANK and RANKL expression on the cells. The results showed that LLDT-8 up-regulated OPG expression on CD3^+^ T leukomonocyte in peripheral blood at doses of 25 nM and 50 nM (Figure 1B), and increased the ratio of OPG/RANKL at the dose of 50 nM (Figure 1C). However, there was no significant difference between RANKL or RNAK expression in the LLDT-8 treated groups and the control group.

**Figure 2:**
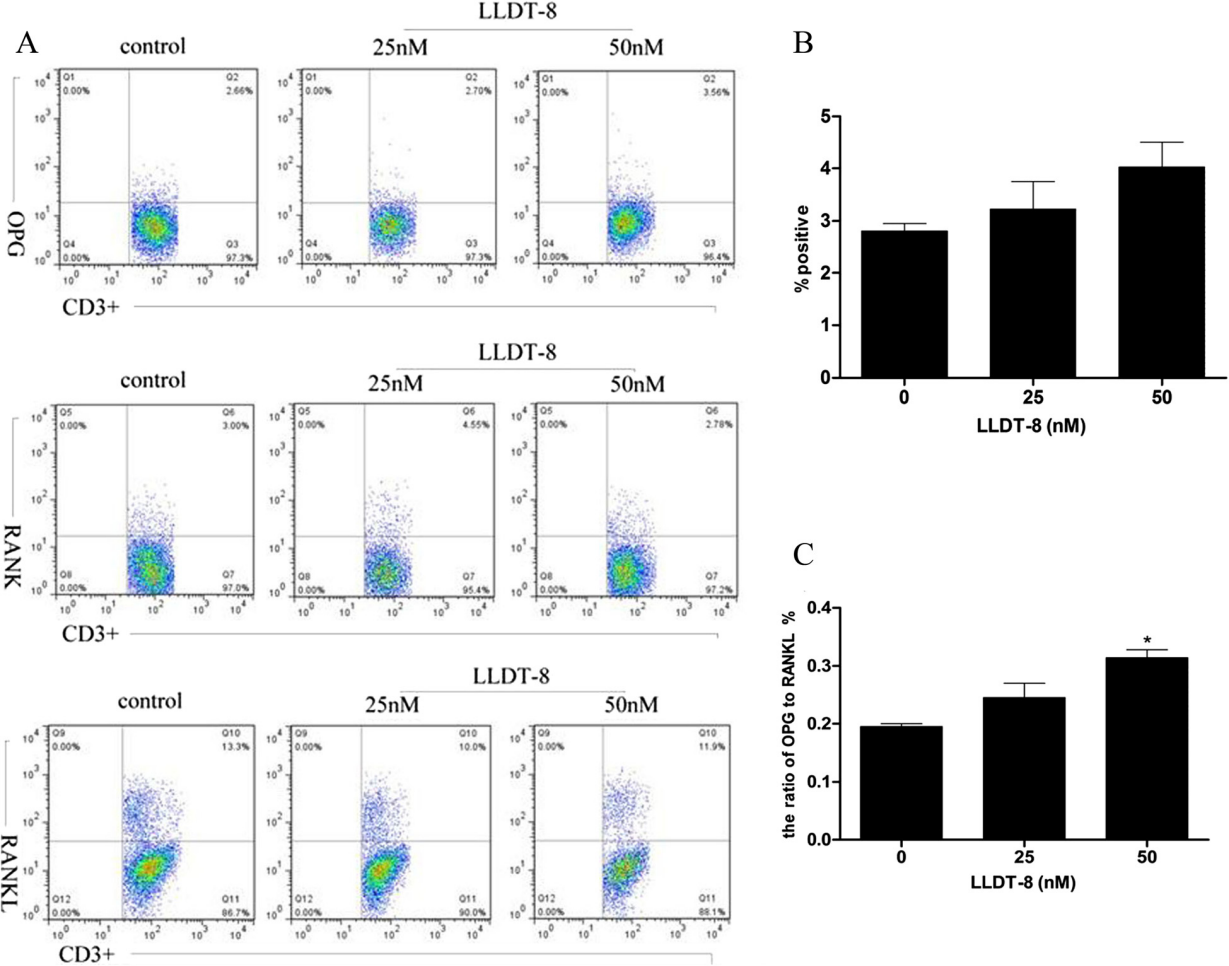
**LLDT-8 increased the ratio of OPG/RANKL in synovial fluid of RA patients.** SFMCs were isolated from synovial fluid of RA patients and treated with various concentrations of LLDT-8 (0, 25 and 50 nM, respectively) for 24 hours, then stained and detected by flow cytometry. **(A)** The psuedocolor plots of OPG, RANK and RANKL. **(B)** The rate of OPG expression on CD3^+^ T leukomonocyte in the three groups. **(C)** The ratio of OPG/RANKL on CD3^+^ T cells in the three groups. *p < 0.05 compared to the control group (0 nM).

We also isolated SFMCs from synovial fluid of RA patients, and examined the OPG, RANK and RANKL expression on these cells treated by the same method as PBMCs. The results showed LLDT-8 (50 nM) increased the ratio of OPG/RANKL on CD3^+^ T cells in synovial fluid (Figure 2C), and there was an upward trend on OPG expression (Figure 2B). However, there was no distinct effect of LLDT-8 on the rate of RANKL or RANK expression on CD3^+^ T cells in synovial fluid.

All these data suggested LLDT-8 may up-regulate OPG expression on CD3^+^ T leukomonocyte in peripheral blood of RA patients, and increase the ratio of OPG/RANKL on CD3^+^ T leukomonocyte in both peripheral blood and synovial fluid.

### LLDT-8 inhibited IL-1β, IL-6, IL-21 and IL-23 secretion, but promoted the secretion of IL-10

**Figure 3:**
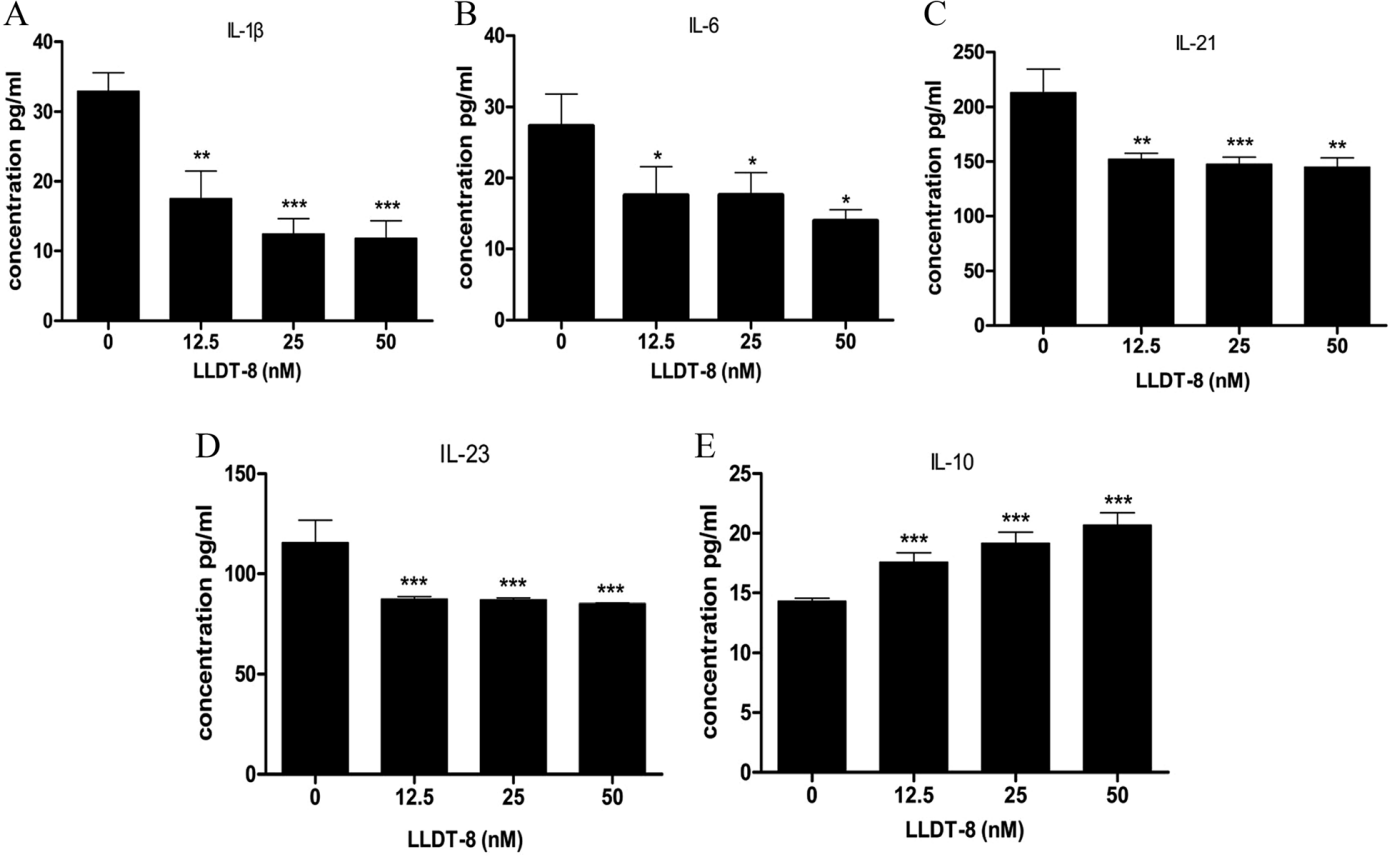
**LLDT-8 inhibited secretion of IL-1β, IL-6, IL-21 and IL-23, but promoted the secretion of IL-10 in the supernatants of PBMCs from peripheral blood of RA patients.** PBMCs were isolated from peripheral blood of RA patients and cultured in the presence of anti-human CD3 (0.4 μg/ml) and LLDT-8 (0, 12.5, 25 and 50 nM, respectively) for 48 hours in incubator. The cell supernatants were harvested, and the levels of the proteins were detected by ELISA. **(A)** The levels of IL-1β in the supernatants of the four groups. **(B)** The levels of IL-6 in the supernatants of the four groups. **(C)** The levels of IL-21 in the supernatants of the four groups. **(D)** The levels of IL-23 in the supernatants of the four groups. **(E)** The levels of IL-10 in the supernatants of the four groups. *p < 0.05, **p < 0.01, ***p < 0.001, compared to the control group (0 nM).

To evaluate the effect of LLDT-8 on the secretion of cytokines in PBMCs from RA patients, the cells were cultured in the presence of anti-human CD3 (0.4 μg/ml) with various concentrations of LLDT-8 (0, 12.5, 25 and 50 nM, respectively) for 48 hours. The cell culture supernatants were harvested, and the levels of cytokines (IL-1β, IL-6, IL-21, IL-23 and IL-10) were assayed by ELISA. The results showed that the concentrations of IL-1β, IL-6, IL-21 and IL-23 in LLDT-8 treated groups were lower than those in the control group. This suggested LLDT-8 can reduce the levels of IL-1β, IL-6, IL-21 and IL-23 in the cell supernatants (Figure 3A-D). Additionally, the concentrations of IL-10 in LLDT-8 treated groups were higher than those in the control group, which indicated that LLDT-8 could increase the level of IL-10 in the cell supernatants (Figure 3E). All these data suggested that LLDT-8 inhibited PBMCs secreting IL-1β, IL-6, IL-21 and IL-23; on the other hand it could promote the secretion of IL-10 in PBMCs.

**Figure 4:**
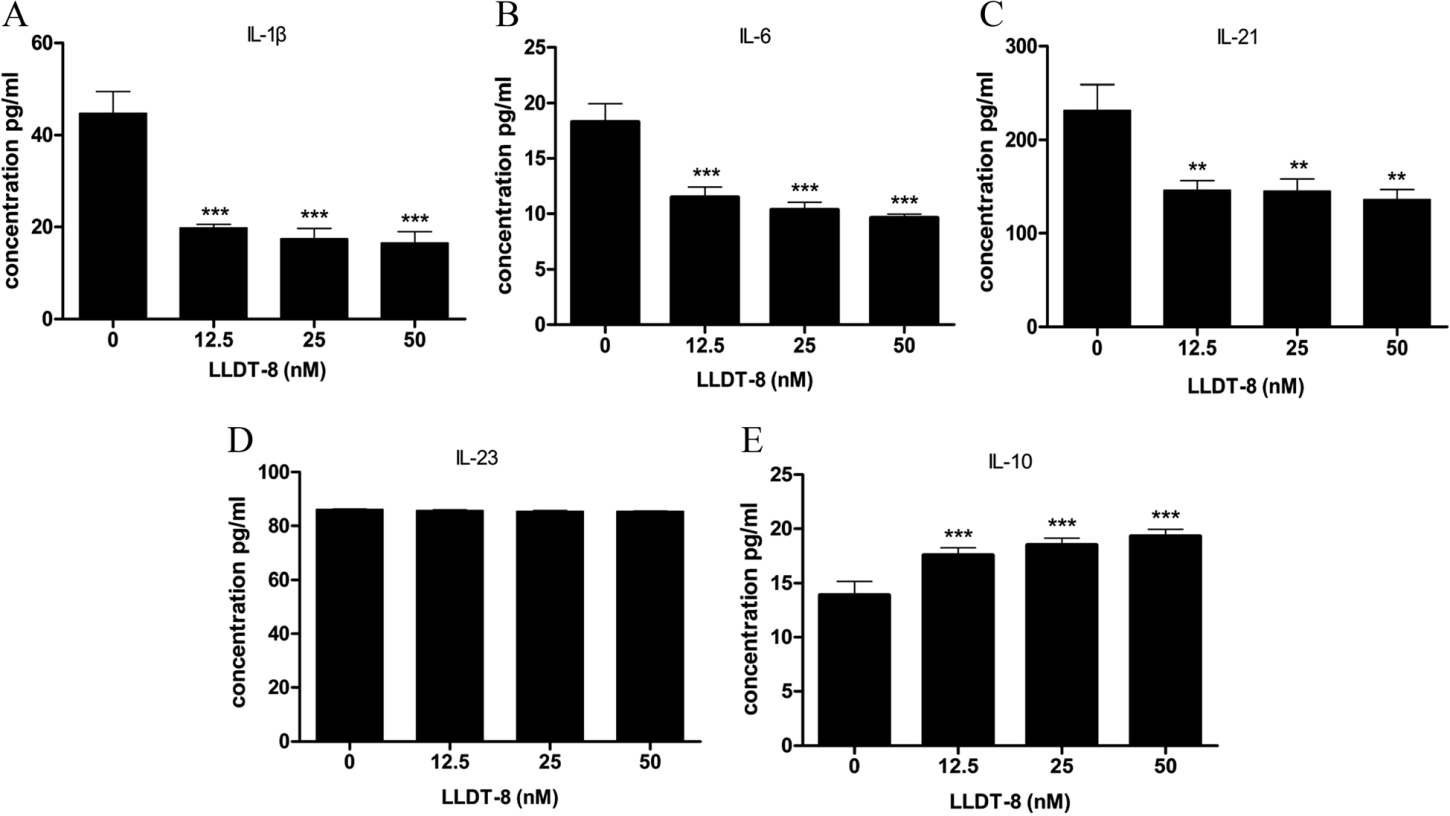
**LLDT-8 inhibited secretion of IL-1β, IL-6 and IL-21, but promoted the secretion of IL-10 in the supernatants of SFMCs from synovial fluid of RA patients**. SFMCs were isolated from synovial fluid of RA patients and cultured in the presence of anti-human CD3 (0.4 μg/ml) and LLDT-8 (0, 12.5, 25 and 50 nM, respectively) for 48 hours in incubator. The cell supernatants were harvested and the levels of the proteins were detected by ELISA. **(A)** The levels of IL-1β in the supernatants of the four groups. **(B)** The levels of IL-6 in the supernatants of the four groups. **(C)** The levels of IL-21 in the supernatants of the four groups. **(D)** The level of IL-23 in the supernatants of the four groups. **(E)** The levels of IL-10 in the supernatants of the four groups. **p < 0.01, ***p < 0.001, compared to the control group (0 nM).

Besides, we also detected the effect of LLDT-8 on the secretion of cytokines in SFMCs from RA patients, and found that LLDT-8 reduced the levels of IL-1β, IL-6, and IL-21 in the supernatants of SFMCs (Figure 4A-C). However, we did not find any distinct effect of LLDT-8 on the secretion of IL-23 (Figure 4D). We also found that LLDT-8 increased the level of IL-10 in the cell supernatants (Figure 4E). Above all, the results demonstrated that LLDT-8 could inhibite SFMCs secreting IL-1β, IL-6 and IL-21, but promoted IL-10 secretion in SFMCs.

### LLDT-8 decreased the number of TRAP-positive cells

**Figure 5:**
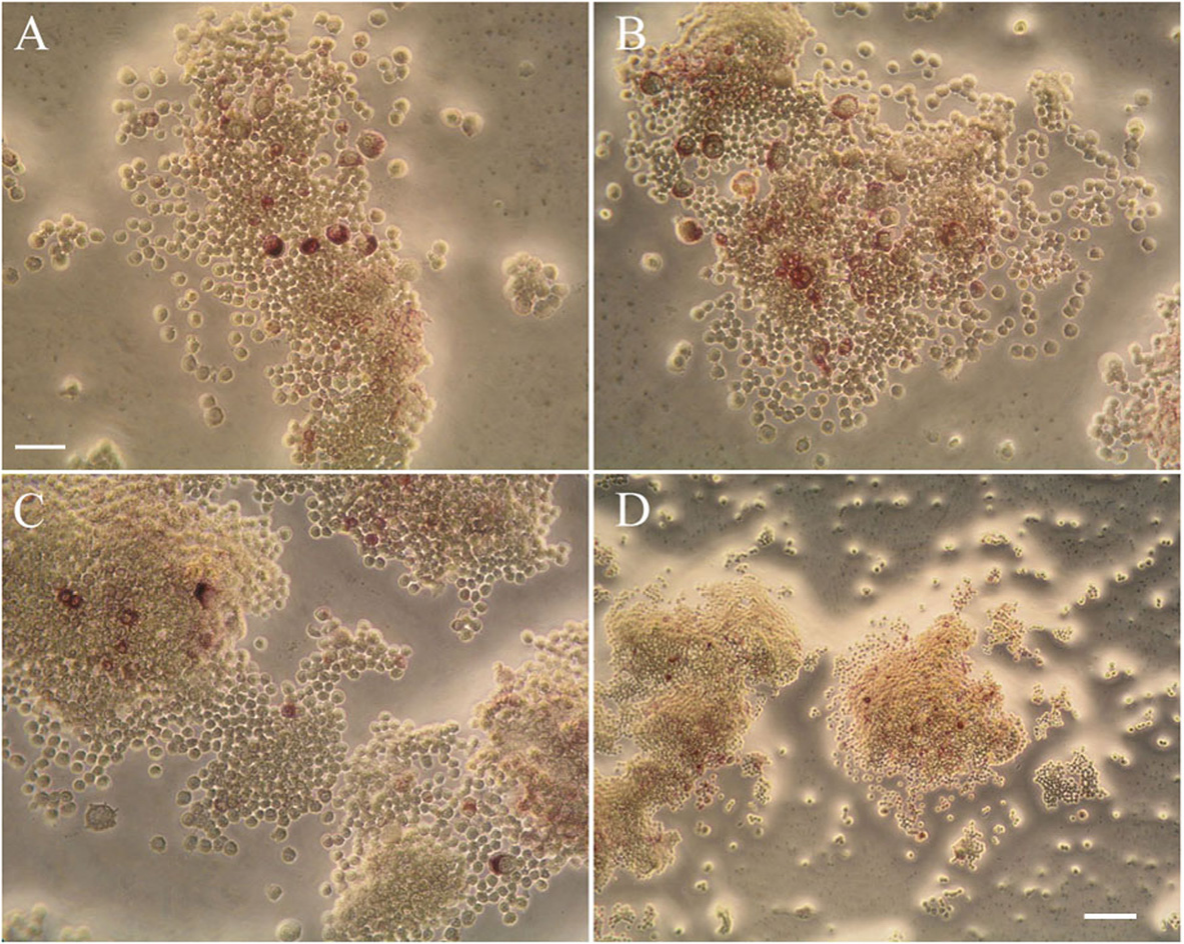
**Cell morphology of RAW264.7.** RAW264.7 cells were cultured in the presence of RANKL (50 ng/ml) and M-CSF (50 ng/ml) with various concentrations of LLDT-8 (0, 12.5, 25 and 50 nM, respectively) for 6 days, then the cells were fixed and stained with TRAP kit on day 1, 2, 3 and 6. The cell morphology were observed using light microscopy, The number of TRAP-positive staining multinuclear cells increased gradually since 72 h **(A)**, and reached the top amount at day 6 **(B)**. A small number of TRAP-positive cells were observed at day 6 in the presence of RANKL (50 ng/ml) and M-CSF (50 ng/ml) with 50 nM LLDT-8 **(C)**. TRAP-positive cells were not observed in the culture medium without RANKL and M-CSF **(D)**. (A-C) 400-fold amplification, bar = 25 μm; **(D)** 200-fold amplification, bar = 50 μm.

**Figure 6:**
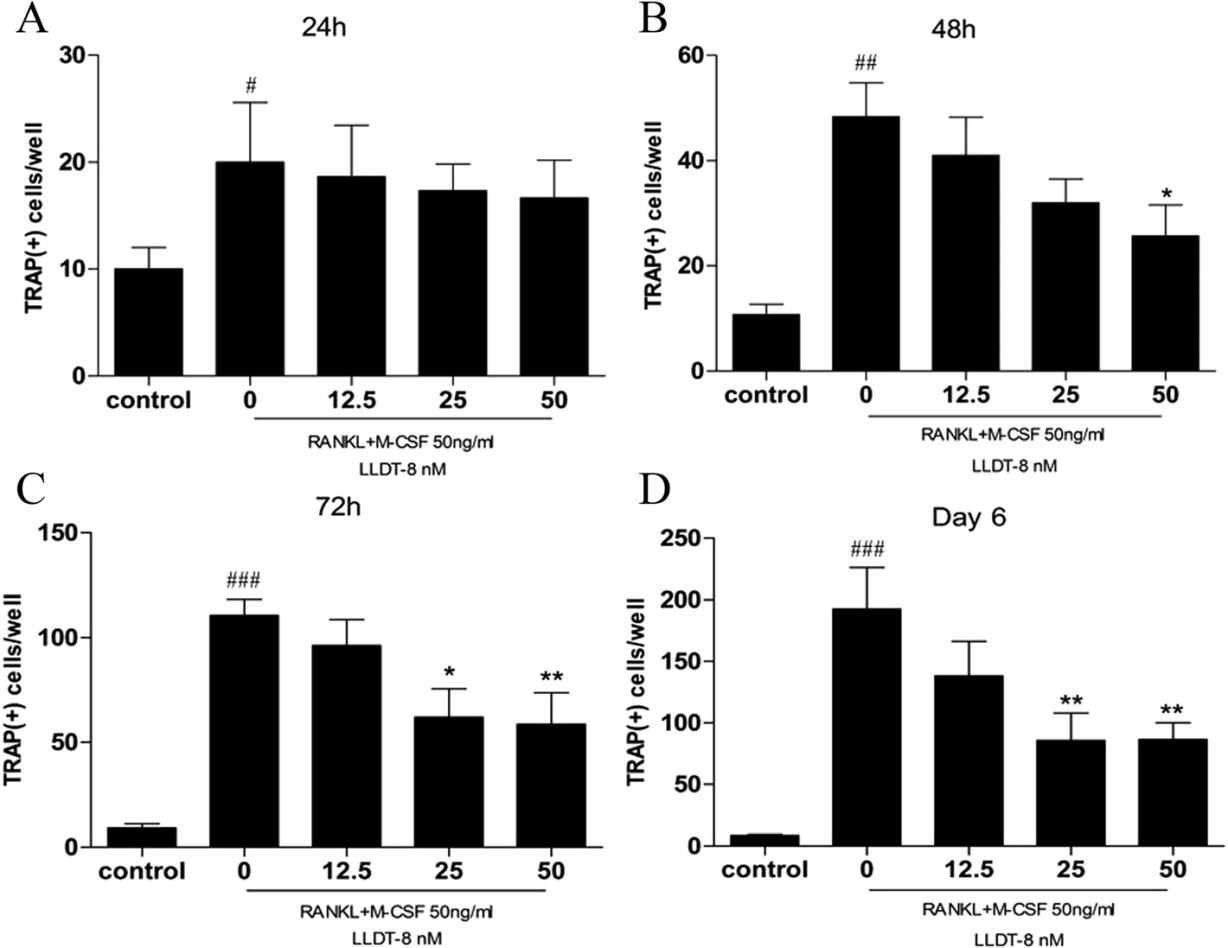
**LLDT-8 decreased the number of TRAP-positive cells.** RAW264.7 cells were cultured in the presence of RANKL (50 ng/ml) and M-CSF (50 ng/ml) with various concentrations of LLDT-8 (0, 12.5, 25 and 50 nM, respectively) for 6 days, the cells of the control group were cultured without RANKL and M-CSF. All the cells were fixed and stained for TRAP according to the manufacturer’s instructions on day 1, 2, 3 and 6. Osteoclast formation was determined to be TRAP-positive staining multinuclear cells using light microscopy. The total number of TRAP-positive cells in each well was counted on day 1 **(A)**, 2 **(B)**, 3 **(C)** and 6 **(D)**, respectively. #p < 0.05, ##p < 0.01, and ###p < 0.001, compared with the control group; *p < 0.05,**p < 0.01, compared with LLDT-8-non-treated (0 nM) group.

To detect the effect of LLDT-8 on RANKL-induced osteoclastogenesis, RAW264.7 cells were cultured in the presence of RANKL (50 ng/ml) and M-CSF (50 ng/ml) with or without various concentrations of LLDT-8 for 6 days. The total number of TRAP-positive cells in each well was counted. We found that the cells showed enlarged volume and irregular shape, and the staining was burgundy. The number of osteoclasts increased gradually since 72 h (Figure 5A), and reached its top amount at day 6 (Figure 5B). A small number of TRAP-positive cells were observed at day 6 in the presence of RANKL (50 ng/ml) and M-CSF (50 ng/ml) with 50 nM LLDT-8 (Figure 5C). The cells without RANKL and M-CSF inducing, were unable to transform into TRAP-positive osteoclasts (Figure 5D). In comparison with the non-treated group, LLDT-8 reduced the number of TRAP-positive osteoclasts at 48 h (Figure 6B), and significantly decreased the number of TRAP-positive osteoclasts from the 3rd day to the 6th day (Figure 6C & D). We did not observe any significant difference between LLDT-8-treated groups and the non-treated group at 24 h (Figure 6A).

### LLDT-8 inhibited RANKL- induced NF-κB activation

**Figure 7:**
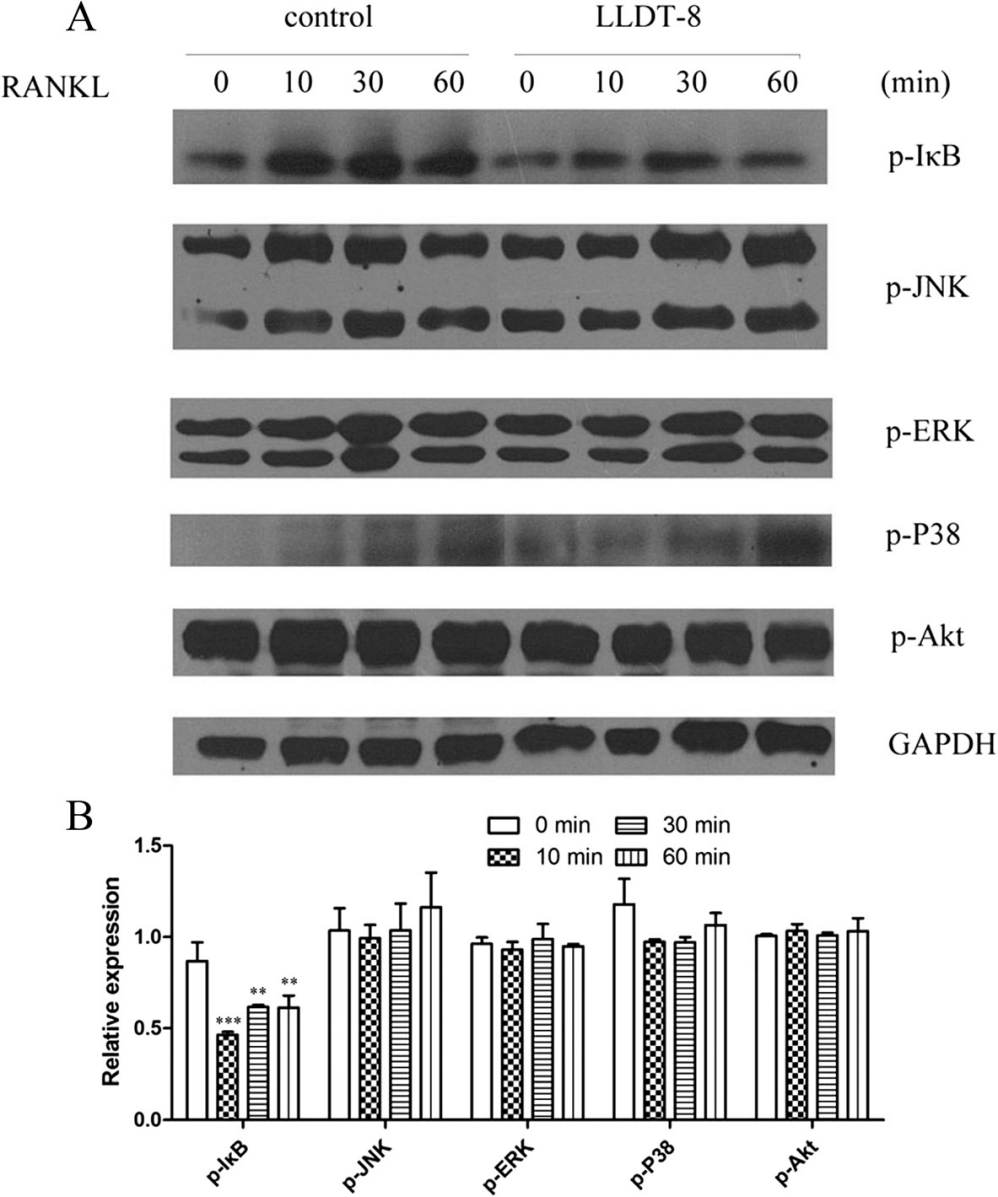
**The effect of LLDT-8 on key proteins of the downstream signaling pathways of RANKL.** RAW264.7 cells were treated with LLDT-8 (0 and 50 nM, respectively) for 4 hours, then induced by culture medium supplemented with 50 ng/ml RANKL for 0, 10, 30 and 60 min, respectively. The protein levels of p-IκB, p-P38, p-JNK, p-ERK and p-Akt were detected by western blotting. **(A)** Immunoblots of the key proteins. RANKL significantly increased the levels of phosphorylation of I-κB, P38, JNK, ERK and Akt in a short time. **(B)** Relative expression of the key proteins. Compared with the control group, LLDT-8 markedly inhibited the expression of p-IκB induced by RANKL. However, it had little effect on the expression of p-P38、p-JNK、p-ERK and p-Akt induced by RANKL. **p < 0.01, ***p < 0.001, compared to the control group (0 nM) at the same time point.

To reveal the mechanism of LLDT-8 on inhibiting osteoclastogenesis, we tested the effect of LLDT-8 on the downstream signaling pathways of RANKL by Western blotting. RANKL significantly increased the levels of phosphorylation of I-κB, P38, JNK, ERK and Akt in a short time (Figure 7A). Compared with the control group, LLDT-8 markedly inhibited the expression of p-IκB induced by RANKL. However, it had little effect on the expression of p-P38、p-JNK、p-ERK and p-Akt induced by RANKL (Figure 7B).

## Discussion

RANK, RANKL and OPG, are essential for the differentiation and activation of osteoclasts. RANKL interacts with its receptor RANK locating on the osteoclast membrane to initiate osteoclastic activation, subsequently induces osteoclasts differentiation and inhibits their apoptosis. OPG acts as an endogenous decoy receptor that competitively inhibits osteoclasts differentiation through its interaction with RANKL [10]. However, the balance of RANKL/RANK/OPG signaling pathway is broken in RA patients. Compared with healthy individuals, RA patients showed significantly higher serum levels of RANKL, lower levels of OPG, and decreased OPG/RANKL ratio in peripheral blood. Moreover, there is a strictly positive interrelation between OPG/RANKL ratio and joint swelling index, as well as health assessment questionnaire (HAQ) [11]. Therefore, to change the ratio of OPG/RANKL might stop or at least slow down the deterioration of this disease.

Our results demonstrated that LLDT-8 up-regulated the OPG expression on CD3^+^ T lymphocytes in peripheral blood of RA patients, leading to the increased OPG/RANKL ratio on CD3^+^ T cells both in peripheral blood and in synovial fluid. Meanwhile, LLDT-8 treatment suppressed the secretion of inflammatory cytokines, such as IL-1β, IL-6, IL-21 and IL-23, but promoted the production of anti-inflammatory cytokine IL-10, in order to regulate RANKL/RANK/OPG system. The ratio of OPG/RANKL was significantly increased by LLDT-8, which indicated that corresponding improvement might be achieved in the patients under LLDT-8 treatment.

The mechanism on which LLDT-8 regulates OPG/RANKL ratio and inhibits osteoclastic activation may involve several aspects. RANKL expression is regulated by various cytokines, such as bone resorbing factor, glucocorticoid, 1α,25(OH)_2_D3, IL-1, IL-6, IL-11, IL-17, TNF-α, PGE2, PTH and so on. They carry out their functions via inducing RANKL expression in osteoblasts, and thus promote bone resorption [12]. IL-21 enhances osteoclastogenesis *in vitro* by up-regulating the expression of RANKL in CD4^+^ T cells in CIA mice as well as CD4^+^ T cells and fibroblast-like FLS of RA patients [13]. Besides, IL-23 also induces RANKL expression in FLS of RA patients through MEK1/2, NF-κB and STAT3 signaling pathway, which was reported to involve in the pathogenesis of RA [14]. The serum level of IL-23 is associated with the symptom of RA. In contrast, IL-10 suppresses RANK-induced osteoclastogenesis and selectively inhibits calcium signaling downstream of RANK by suppressing the transcription of TREM-2 [15]. IL-4 also serves as a protective cytokine in RA, which inhibits osteoclasts formation by stimulating osteoblasts to produce OPG [16]. These studies confirmed that pro-inflammatory cytokines, IL-1, IL-6, IL-17, IL-21, IL-23 and TNF-α induce osteoclasts formation via RANKL signaling pathway; on the other hand, anti-inflammatory cytokines like IL-4 and IL-10 block RANKL signaling pathway and in turn inhibit osteoclasts formation. In our study, we showed that LLDT-8 enhanced the ratio of OPG/RANKL by increasing OPG expression and down-regulated the production of IL-1β, IL-6 and IL-21 in PBMCs and SFMCs. LLDT-8 also suppressed the production of IL-23 in PBMCs, but promoted IL-10 secretion in PBMCs and SFMCs. Previous studies reported that LLDT-8 inhibited TNF-α and IL-17 secretion in PBMCs and SFMCs but up-regulated IL-4 expression in CD4^+^ T cells. All these results suggested that LLDT-8 probably regulated RANKL/RANK/OPG system by up-regulating the expression of osteoclasts inhibitive factors in RA patients. These data also explained how LLDT-8 exerts its function on anti-osteoclastogenesis in RA patients.

Up to now, it is not easy to collect mature osteoclasts from animal or human tissues. The osteoclasts required were usually obtained from differentiation of osteoclastic progenitor. Recently, methods of inducing neoblast to differentiate into osteoclast *in vitro* is well established, for example, co-culturing marrow stroma cell with myelomoncyte, stimulating myelomoncyte with culture medium of marrow stroma cell, and inducing myelomoncyte and mice RAW264.7 cell with mice recombination RANKL. Among them, the osteoclasts induced by RANKL were larger in both volume and number. This method was easier to operate compared with other methods [17]. RAW264.7 is mice mononuclear macrophage cell line, and can differentiate into osteoclast-like cells in the presence of RANKL and M-CSF. During our research we obtained osteoclasts easily and steadily by using this method. Our results showed that RAW264.7 cells differentiated into TRAP-positive osteoclast-like cells in the presence of RANKL and M-CSF, and LLDT-8 decreased the osteoclastogenesis. To reveal its mechanism of action, we further examined the signaling pathway involved in RANK/RANKL/OPG system under LLDT-8 treatment. NF-κB plays an essential role in the process of osteoclasts differentiation and anti-apoptosis. NF-κB p50 and p52 expression is essential for RANK-expressing osteoclast precursors to differentiate into TRAP-positive osteoclasts in response to RANKL and other osteoclastogenic cytokines [18]. Interestingly, we found that LLDT-8 inhibits the expression of p-IκB which could be induced by RANKL. This indicates LLDT-8 probably decreases RANKL-induced osteoclastogenesis by suppressing NF-κB signaling pathway.

Several signaling molecules that promote the differentiation and maturation of osteoclasts participate in RANKL signaling pathway. Mitogen activated protein kinase (MAPK) is one of the serine/threonine phosphorylating kinases. There are many forms of MAPK, including extracellular regulated kinase (ERK), Jun N-terminal kinase/stress-activated protein kinase (JNK/SAPK) p38 MAPK [19], and etc. These kinases are activated in RANKL downstream, and promote osteoclasts differentiation. M-CSF facilitates RANKL-induced activation of c-fos and ERK1/2 phosphorylation, in turn increases the activity of osteoclasts. Studies showed that the function of M-CSF could be blocked by MEK inhibitor PD98059 [20]. As c-Jun is an essential transcription factor for osteoclast differentiation *in vivo*, blockade of the JNK and c-Jun pathway by SP600125 suppresses osteoclast differentiation. In addition, RANKL-induced osteoclasts differentiation is markedly inhibited by over-expression of dominant-negative JNK1 or dominant-negative c-Jun [21]. Bone resorption area and number of osteoclasts *in vivo* were significantly decreased by the treatment of FR167653, a P38 MAPK inhibitor [22]. Phosphatidylinositol-3 kinase (PI3K) plays an important role in multiple biological processes such as proliferation, survival, migration, secretion and etc. The activity of PI3K is regulated by RANK through upstream Src, which is an intracytoplasm protein kinase. Then Akt is activated, which further facilitates osteoclasts differentiation. When RAW264.7 and macrophage precursor cells (BMMs) were treated with RANKL, a higher steady-state level of IP3 was observed in RAW264.7 cells. In BMMs, the inhibition of phospholipase C (PLC) with U73122, a specific inhibitor of PLC and IP3Rs, suppressed the generation of RANKL-induced multinucleated cells and decreased the bone-resorption rate [23]. In our study, significant effect of LLDT-8 on the expression of p-P38, p-JNK, p-ERK and p-Akt induced by RANKL were not observed. Real-time PCR should be used to further reveal the role of LLDT-8 on the downstream signaling pathways of RANKL, including NF-κB pathway. Moreover, how LLDT-8 affects NF-κB pathway is still not clear. Thus, further research needs to be done, in order to clarify the effect of LLDT-8 on the downstream signaling pathways of RANKL.

## Conclusions

In conclusion, our results showed that LLDT-8 plays an important role on anti-osteoclastogenesis effect in RA probably through regulating RANKL/RANK/OPG system and its downstream signaling pathway as well as cytokine productions. However, more details of the mechanisms of the multi-function of LLDT-8 still need to be studied. There may be a prospect of applying LLDT-8 in the treatment of RA.

## Abbreviations

ANOVA: One-way analysis of variance
BMMs: Macrophage precursor cells
ERK: Extracellular regulated kinase
IL: Interleukin
JNK: Jun N-terminal kinase
MAPK: Mitogen activated protein kinase
OPG: Osteoprotegerin
PBMCs: Peripheral blood mononuclear cells
PI3K: Phosphatidylinositol-3 kinase
PLC: Phospholipase C
RA: Rheumatoid arthritis
RANK: Receptor activator of nuclear factor κ-B
RANKL: Receptor activator of nuclear factor κ-B ligand
SAPK: Stress-activated protein kinase
SFMCs: Synovial fluid mononuclear cells
TRAP: Tartaric acid phosphatase
